# Bile Salt Hydrolase Activities: A Novel Target to Screen Anti-*Giardia* Lactobacilli?

**DOI:** 10.3389/fmicb.2018.00089

**Published:** 2018-02-08

**Authors:** Thibault Allain, Soraya Chaouch, Myriam Thomas, Marie-Agnès Travers, Isabelle Valle, Philippe Langella, Philippe Grellier, Bruno Polack, Isabelle Florent, Luis G. Bermúdez-Humarán

**Affiliations:** ^1^INRA, Commensal and Probiotics-Host Interactions Laboratory, Micalis Institute, AgroParisTech, Paris, France; ^2^UMR 7245, Muséum National d’Histoire Naturelle, Centre National de la Recherche Scientifique, Sorbonne Universités, Paris, France; ^3^INRA, Ecole Nationale Vétérinaire d’Alfort, BIPAR, ENVA, ANSES, UMR, Université Paris-Est, Champs-sur-Marne, France; ^4^INRA, Laboratoire de Santé Animale, BIPAR, ENVA, ANSES, UMR, Maisons-Alfort, France

**Keywords:** *Giardia duodenalis*, lactobacilli, *Lactobacillus johnsonii*, *Lactobacillus gasseri*, probiotics, bile salt hydrolases

## Abstract

*Giardia duodenalis* is a protozoan parasite responsible for giardiasis, a disease characterized by intestinal malabsorption, diarrhea and abdominal pain in a large number of mammal species. Giardiasis is one of the most common intestinal parasitic diseases in the world and thus a high veterinary, and public health concern. It is well-established that some probiotic bacteria may confer protection against this parasite *in vitro* and *in vivo* and we recently documented the implication of bile-salt hydrolase (BSH)-like activities from strain La1 of *Lactobacillus johnsonii* as mediators of these effects *in vitro*. We showed that these activities were able to generate deconjugated bile salts that were toxic to the parasite. In the present study, a wide collection of lactobacilli strains from different ecological origins was screened to assay their anti-giardial effects. Our results revealed that the anti-parasitic effects of some of the strains tested were well-correlated with the expression of BSH-like activities. The two most active strains *in vitro*, La1 and *Lactobacillus gasseri* CNCM I-4884, were then tested for their capacity to influence *G. duodenalis* infection in a suckling mice model. Strikingly, only *L. gasseri* CNCM I-4884 strain was able to significantly antagonize parasite growth with a dramatic reduction of the trophozoites load in the small intestine. Moreover, this strain also significantly reduced the fecal excretion of *Giardia* cysts after 5 days of treatment, which could contribute to blocking the transmission of the parasite, in contrast of La1 where no effect was observed. This study represents a step toward the development of new prophylactic strategies to combat *G. duodenalis* infection in both humans and animals.

## Introduction

*Giardia duodenalis* (also known as *G. lamblia* or *G. intestinalis*) is the etiologic agent of the zoonotic disease giardiasis, one of the most common waterborne parasitic infections globally. *G. duodenalis* is a flagellate protozoan (Excavata, Diplomonad) that infects a broad diversity of animals such as humans, mammals, reptiles and birds (Thompson and Monis, 2004, 2012). Transmission can occur by ingestion of viable cysts from contaminated water and soil, or directly by contact with an infected animal’s feces (Gardner and Hill, 2001). After ingestion, exposure to gastric acid and proteases in the stomach leads to excystation and liberation of replicative trophozoite stages that adhere transiently to the proximal small intestine and persist for several days (for up to several months in some cases) (Reiner et al., 2003). After this replicative and pathogenic stage, trophozoites are carried into the colon and progressively encyst. Cysts, released through host feces, may then survive in the environment for several months and remain infectious at low doses (Thompson et al., 1993).

The main clinical symptoms of giardiasis are acute or chronic diarrhea, abdominal pain, intestinal malabsorption, steatorrhea, and weight loss (Farthing, 1996; Buret, 2007). Considered as a public health threat and a veterinary concern worldwide, giardiasis is responsible for many waterborne diarrhea outbreaks in developed countries (Roxstrom-Lindquist et al., 2006; Buret, 2007). In humans, it affects mainly children, and undernourished or immunosuppressed individuals. Even though giardiasis can be self-limited, standard antibiotic therapies including 5-nitroimidazole and benzimidazole drugs are needed to treat long-term infections (Savioli et al., 2006). In mammals, *G. duodenalis* infections are common in companion animals, small ruminants and cattle, for which alternative treatments are very limited (Harris et al., 2001).

The increasing number of clinical trial failures and the emergence of resistant *Giardia* strains in both medical and veterinary applications have encouraged the development of new therapeutic strategies (Harris et al., 2001; Upcroft and Upcroft, 2001). In this context, it has been established that gut microbiota plays a pivotal role in the protection against enteropathogens through the production of antimicrobial compounds and by competition for nutrients and attachment to the mucosal surface (Travers et al., 2011; Bengmark, 2013). In particular, probiotic bacteria such as some strains of lactobacilli have been shown to confer host health benefits by enhancing innate and adaptive immune responses (Sanders, 2008; Martin et al., 2013; Sokol, 2014). Moreover, pre-clinical studies suggest that colonization of the small intestine by *G. duodenalis* trophozoites depends on the composition of host gut microbiota (Singer and Nash, 2000; Barash et al., 2017; Bartelt et al., 2017). In recent years, probiotic-based therapies have been explored to treat giardiasis (Allain et al., 2017). For instance, some lactobacilli strains such *Lactobacillus johnsonii* La1 (also known as NCC533 but hereafter named La1), *Lactobacillus casei* MTCC1423 and *Lactobacillus rhamnosus* GG (LGG), have been shown to display anti-giardial properties by (i) antagonizing the proliferation of trophozoites and, (ii) reducing the severity of infection in several murine models (Humen et al., 2005; Shukla et al., 2008; Goyal et al., 2011; Travers et al., 2011). While the molecular mechanisms remain poorly understood, we have recently discovered the involvement of deconjugated bile salts, generated by the hydrolysis of conjugated bile salts from bile by La1 strain, as one of the mechanisms contributing to the inhibition of *Giardia* trophozoite growth *in vitro* (Perez et al., 2001; Travers et al., 2016). Our hypothesis was that these deconjugated bile salts were produced by bile salt hydrolase (BSH)-like enzymes released or secreted by this strain (Travers et al., 2016).

In the current study, we screened a wide collection of lactobacilli strains from various environmental origins in order to determine their ability to display an anti-giardial effect. *In vitro* analysis from supernatant-lactobacilli samples led to the identification of several strains having a strong inhibitory activity against *G. duodenalis* growth (up to the level previously reported for La1). In parallel, we showed that the *in vitro* anti-*Giardia* activity displayed by some of these lactobacilli strains is clearly correlated with their BSH-like activities *in vitro*. The two strains displaying the strongest inhibitory effects: La1 and *Lactobacillus gasseri* CNCM I-4884, were then evaluated *in vivo* in a suckling mice model challenged with the WB6 strain of *G. duodenalis*. Our results showed that *L. gasseri* CNCM I-4884 displayed a higher anti-giardial effect *in vivo* than La1, with an almost complete clearance of *Giardia* infection in suckling mice. These findings show that a screening based on the detection of BSH activities is a promising tool to identify new anti-*Giardia* lactobacilli strains. Overall, our study opens new therapeutic strategies for both preventing and treating giardiasis in humans.

## Materials and Methods

### *Giardia duodenalis* Culture Conditions

*Giardia duodenalis* was grown *in vitro* as recently described (Travers et al., 2016). Trophozoites of *G. duodenalis* strain WB clone 6 (WB6) assemblage A1 (ATCC 50803) were grown in Keiser’s modified TYI-S-33 medium (KM) (Morrison et al., 2007). KM medium was supplemented with 10% heat-inactivated fetal calf serum (FCS, reference A15-101, PAA Laboratories, GE healthcare), adjusted to pH 6.0, and sterilized with a 0.22 μm filter. *G. duodenalis* WB6 trophozoites were subcultured in anaerobic conditions at 5 × 10^4^ cells per ml after chilling on ice for 10 min and centrifuged at 700 ×*g*, 5 min. Aliquots were frozen in liquid nitrogen and stored at -80°C until further use. For *in vitro* and *in vivo* experiments, 48 h old cultures of confluent trophozoites were pelleted at 700 × *g*, 5 min after chilling on ice for 10 min, and resuspended at the needed concentrations.

### Bacterial Strains and Culture Conditions

The bacteria collection used in this study comprises 29 lactobacilli isolated from different biotopes (**Table 1**). The strains were grown in Man Rogosa Sharpe (MRS, Difco) medium on agar plates over night at 37°C in anaerobic conditions (GasPack Plus, BBL). Bacteria were then subcultured for 16 h at 37°C in MRS broth and were subsequently grown in Keiser’s modified TYI-S-33 medium supplemented with 10% heat-inactivated FCS (see above) to stationary phase. An intermediary subculture in modified TYI-S-33 medium was used for some strains as previously described (Perez et al., 2001). Bacterial supernatants were then collected after centrifugation at 10,000 ×*g* for 10 min, sterilized with a 0.22 μm filter and the pH was adjusted to 6.2 with 5N NaOH.

**Table 1:** List of tested lactobacilli strains with their characteristics and bile salt hydrolase activity specificities.

*Lactobacillus* species	Strain^1^	Origin	TDCA (0.5%) hydrolase activity	TDCA hydrolase activity score^2^	GDCA (0.5%) hydrolase activity	GDCA hydrolase activity score^2^
*L. acidophilus*	ATCC4356	Feces, human	P	+	N	-
*L. brevis*	CNRZ1845	Malt (beer)	P	+	P	+
*L. casei*	ATCC393	Cheese	N	-	N	-
*L. crispatus*	CIP103606	Unknown	N	-	P	++
*L. curvatus*	CNRZ1335	Fermented sausage/mea	N	-	N	-
*L. gasseri*	ATCC33323	Vaginal tract, human	N	-	N	-
*L. gasseri*	CNCM I-4884 ATCC29601	Cariouth tooth, human	P	+++	P	++
*L. helveticus*	CNRZ1109	Lactic starter	N	-	N	-
*L. helveticus*	ATCC11977	Lactic starter (cheese)	N	-	P	++
*L. johnsonii*	CNRZ217	Feces, rat	N	-	P	+
*L. johnsonii*	CNRZ218	Feces, rat	P	+	P	++
*L. johnsonii*	CNRZ1897	Fermented milk	P	+++	ND	ND
*L. johnsonii*	CIP103614	Vaginal tract, human	P	++	N	-
*L. johnsonii*	CIP103786	Cheese	P	+	P	++
*L. johnsonii*	CIP103652	Unknown	P	++	N	-
*L. johnsonii*	CIP103653	Unknown	P	++	N	-
*L. johnsonii*	CIP103654	Pharmaceutical preparation	P	+	P	+
*L. johnsonii*	CIP103781	Unknown	P	+++	P	++
*L. johnsonii*	CIP103782	Urethran, human	P	+	N	-
*L. johnsonii*	ATCC33200	Blood, human	P	++	P	++
*L. johnsonii*	La1 (NCC533)	Feces, human	P	+++	P	++
*L. paracasei*	CNRZ315	Unknown	N	-	N	-
*L. pentosus*	CNRZ1218	Cheese	N	-	N	-
*L. plantarum*	CNRZ738	Silage	N	-	P	+
*L. rhamnosus*	CNRZ317	Unknown	N	-	N	-
*L. rhamnosus*	CNRZ2084	Fermented milk	N	-	N	-
*L. reuteri*	CNRZ431	Sourdough	N	-	N	-
*L. sakei*	CNRZ1332	Moto, starter of sake	N	-	N	-
*L. zeae*	CNRZ2269	Corn steep liquor	N	-	N	-


*P, positive; N, negative; ND, non-determined. ^*1*^Strains were obtained from different collections: CIP, Collection de l’Institut Pasteur, France; CNRZ/CIRM, Centre International de Ressources Microbiennes; CNCM, Collection Nationale de Cultures de Microorganismes, Institut Pasteur, France; ATCC, American Type Culture Collection, United States. ^*2*^TDCA/GDCA hydrolase activity score were determined on the size of the halo zone (diameter): (-): no detected halo; (+): <8 mm; (++): 9 mm to 12 mm; (+++): >13 mm.*

### Bile Salt Hydrolase Assays

The bacterial strains were tested for Tauro-deoxycholic acid (TDCA) and Glyco-deoxycholic acid (GDCA) hydrolase activities on MRS-agar plates supplemented with either 0.5% TDCA (Sigma–Aldrich) or 0.5% GDCA (Merck) following the protocol described by Moser and Savage (2001). MRS plates with 0.5% TDCA were first incubated at 37°C in anaerobic conditions for 24 h prior to inoculation. Strains were grown anaerobically for 16 h at 37°C in MRS broth, then streaked on MRS-agar plates, supplemented or not, with 0.5% TDCA or 0.5% GDCA and incubated at 37°C in anaerobic conditions for 48–72 h. BSH activity can be easily detected when unconjugated deoxycholic acid precipitates in the MRS-agar plate, forming an iridescent halo below and around active colonies. These assays were performed in duplicates, and included two biological replicates. BSH activity score was determined as indicated in **Table 1**.

### *In Vitro* Anti-giardial Assays

Filtered bacterial supernatants (500 μl) were co-incubated with fresh cultures of *Giardia* trophozoites (1.33 × 10^5^ parasites/ml in KM medium, pH 6.0, supplemented with 10% heat-inactivated FCS) at a volumetric ratio of 1–3, in the presence or absence of bovine bile (0.6 g/L) at 37°C in anaerobic conditions for 22 h. BSH from *Clostridium perfringens* (1 U) (reference C4018, Sigma–Aldrich) was used as a positive control for *Giardia* growth inhibition as previously described (Travers et al., 2016). Samples were then ice-chilled for 10 min and trophozoite load was determined using hemocytometer (flagella mobility was used to screen parasite viability). The inhibition rates were determined by counting the number of living trophozoites and establishing ratios compared to the controls (in percentage). Experiments were conducted in duplicates and included three biological replicates.

### Assessment of Anti-giardial Activity*in Vivo*

Cultures of *G. duodenalis* trophozoites WB6 were grown in Keister’s modified TYI-S-33 medium with 10% heat-inactivated FCS. Lactobacilli strains (La1, *L. gasseri* CNCM I-4884 and *L. curvatus* CNRZ1335) were cultured in MRS broth for 16 h at 37°C in anaerobic conditions from precultures. Bacteria were then harvested by centrifugation at 7000 ×*g* for 15 min, washed two times and resuspended in a corresponding volume of sterile PBS/Glycerol 15% to obtain a final concentration of 2.5 × 10^10^ CFU/ml for intragastric administrations (5 × 10^8^ CFU per neonatal mouse). Mice used for reproduction were non-inbred mice of the OF1 strain (Charles River, Saint-Germain-Nuelles, France). All experiments were conducted in a filtered air chamber and manipulations were performed under a laminar flow hood to prevent contamination of the environment by cysts of *G. duodenalis* and other pathogens.

Lactobacilli strains were administered daily by intragastric gavage in a volume of 20 μl (5 × 10^8^ CFU) to 5 days old neonatal mice from day 5 to day 15 (*n =* 9–12 per group) (**Figure 2**). Control animals (*n* = 8) received PBS with glycerol 15% instead of bacterial suspensions. Neonatal mice were then challenged with trophozoites at day 10 by intragastric gavage in a volume of 100 μl (10^5^ trophozoites). Mice were sacrificed by cervical dislocation at day 16. The small intestine and colon contents were examined for the presence of trophozoites and cysts, by counting the parasite burden, using a hemocytometer. The counting of trophozoites was performed from the small intestine (resuspended in 5 ml of ice-chilled sterile PBS), while cysts were enumerated in the colon and caecum (resuspended in 5 ml of ice-chilled 2.5% Potassium dichromate). The minimum threshold of counting values for statistical analysis was set at 10^3^ trophozoites.

### Statistical Analysis

Results were expressed as means ± standard error of the mean (SEM). Comparison between groups was assessed by one or two-way analysis of variance (ANOVA), *t*-test, Mann–Whitney and Kruskal–Wallis. Correlation tests were performed using Spearman’s Rank Correlation Test. Statistical significance was calculated at *p*-values under 0.05 at 95% confidence interval.

### Ethics Statement

All protocols were carried out in accordance with the institutional ethical guidelines of the ethics committee ANSES’s Animal Health Laboratory at Maisons-Alfort on the campus of the French National Veterinary School of Alfort (ENVA), which approved this study.

## Results

### BSH Activities of Lactobacilli Strains

**FIGURE 1:**
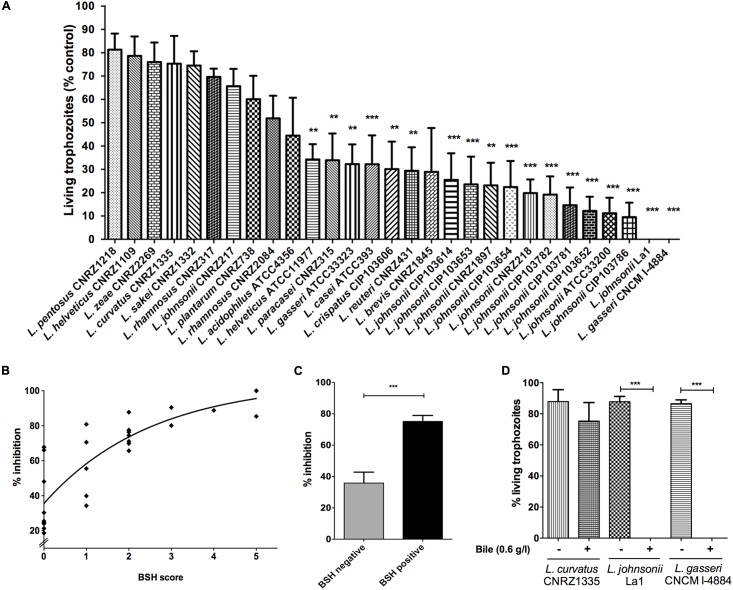
Bile-salt hydrolase (BSH) and anti-*Giardia in vitro* activities of different lactobacilli strains. **(A)** Percentage of living *Giardia duodenalis* trophozoites when cultivated 22 h with lactobacilli supernatants. *G. duodenalis* trophozoites were enumerated after 22 h of co-incubation at 37°C in anaerobic conditions (values are represented with bile supplementation). Values are mean ± SEM. **(B)** Spearman’s rank correlation test between percentage of inhibition of lactobacilli strains and their BSH activity score. BSH score was determined depending on the size of the halo zone (+ = 1; ++ = 2; +++ = 3, **Table 1**) and the ability to deconjugate either tauro-conjugated bile salts or glyco-conjugated bile salts or both (addition of BSH score for each substrate specificity). A positive correlation is observed (*r* = 0.86; *p* < 0.0001). **(C)** Inhibition assays according to Bile Salt Hydrolase activities. Lactobacilli strains were divided into two groups: (i) BSH negative (strains with no detected BSH activity), (ii) BSH positive (strains exhibiting either TDCA, GDCA, or both BSH activities). *G. duodenalis* trophozoites were enumerated after 22 h of co-incubation at 37°C in anaerobic conditions (values are represented with bile supplementation). Values are in mean ± SEM. **(D)** Percentage of living *G. duodenalis* trophozoites when co-cultivated 22 h with lactobacilli strains supernatants, with or without bile supplementation (bovine bile 0.6 g/L). *G. duodenalis* trophozoites were enumerated after 22 h of co-incubation at 37°C in anaerobic conditions. Values are mean ± SEM. ^∗∗^*p* ≤ 0.01; ^∗∗∗^*p* ≤ 0.001.

The 29 strains tested in this study were able to grow in MRS-agar supplemented with 0.5% TDCA whereas only 25 grew in MRS-agar supplemented with 0.5% GDCA, supporting that GDCA has a higher bactericidal activity than TDCA *in vivo* (Begley et al., 2006). TDCA/GDCA hydrolase activity is a well-recognized indicator of BSH activity in bacteria (Bustos et al., 2012). A semi-quantitative method was used to monitor the BSH activity by measuring the halo zone of positive strains after 48–72 h of incubation. Among the 29 strains, 6 exhibited TDCA hydrolase activity, 4 exhibited GDCA hydrolase activity and 8 displayed both TDCA and GDCA hydrolase activities (**Table 1**). No halo was detected for 11 strains. Interestingly, all *L. johnsonii* strains tested displayed at least one type of BSH activity. Among the strains harboring both tauro and glyco-specific BSH activities, La1, *L. gasseri* CNCM I-4884 and *L. johnsonii* CIP103782 exhibited the highest BSH scores (**Table 1** and **Figure 1A**).

### Characterization of Anti-giardial Lactobacilli Strains

We screened the 29 lactobacilli strains for their inhibitory abilities against *G. duodenalis*. For this, bacterial supernatants were co-incubated for 22 h at 37°C with *Giardia* trophozoite cultures in KM growth medium supplemented, or not, with bovine bile (0.6 g/L). Living trophozoites were enumerated using hemocytometer. Parasite cultures that were grown without bile did not display major differences when compared to trophozoites grown in KM supplemented with bile, showing that cholesterol and lipids from fetal bovine serum are sufficient to fulfill the lipid uptake requirements of *Giardia* and that bile components are not toxic for *Giardia* (Travers et al., 2016).

When co-incubated with *Giardia* in KM supplemented with bovine bile, 19 bacterial supernatants exhibited significant antagonistic effects on *Giardia* growth, with a wide range of inhibition levels (**Figure 1A**). Six strains showed growth inhibition levels from 15 to 30%, 10 exhibited growth inhibition levels from 30 to 70%, and 12 strains displayed a strong reduction of trophozoites by 70–100% (*p* < 0.001). Only supernatant of *L. gasseri* CNCM I-4884 were as efficient as that of La1 to antagonize *Giardia* growth *in vitro* (100%). Using Spearman’s Rank Correlation Test, we observed a positive correlation between inhibition levels and BSH activity score (*r* = 0.86; *p* < 0.0001) of bacterial strains (**Figure 1B**). Inhibition assays were then divided into two groups: (i) BSH negative (strains with no detected BSH activity) and (ii) BSH positive (strains displaying either TDCA, GDCA, or both BSH activities). We observed that *Giardia* growth inhibitory activity of BSH positive strains were twofold higher relative to BSH negative strains (Student’s *t*-test; *p* < 0.0001) (**Figure 1C**). Moreover, the cytotoxic effect on *Giardia* was lost when trophozoites were co-incubated with bacterial supernatant in the absence of bile (**Figure 1D**). However, a very weak inhibition was still observed for a majority of the strains (range between 5 and 15%), which can be explained by the presence of other extracellular compounds released by lactobacilli (e.g., bacteriocins) that are also potentially deleterious to *Giardia* growth. Taken together, these *in vitro* results strongly suggest that the anti-giardial effect displayed by lactobacilli strains tested in this study is mostly bile-dependent.

### *In Vivo* Effect of Orally Administered Lactobacilli against *G. duodenalis*

**FIGURE 2:**
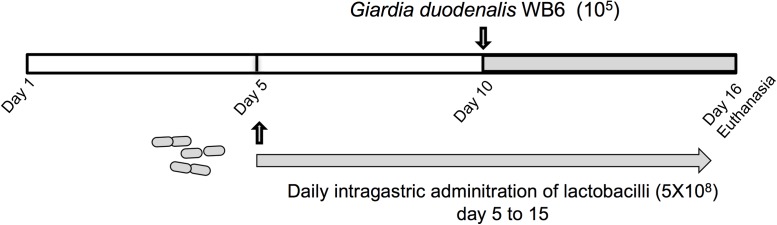
Experimental design of *G. duodenalis* infection in OF1 suckling mice model. Lactobacilli strains were administered daily by intragastric gavage (5 × 10^8^ CFU) to 5 days old OF1 suckling mice from day 5 to day 15. Control animals received PBS/glycerol 15%. Mice were challenged with *G. duodenalis* WB6 trophozoites (10^5^) at day 10 by intragastric gavage. Mice were sacrificed by cervical dislocation at day 16.

*In vivo* experiments were performed in order to assess the potential of newly identified anti-giardial lactobacilli strains to antagonize *G. duodenalis in vivo*. Bacterial suspensions, or PBS/glycerol were daily administered by intragastric gavage (5 × 10^8^ CFU) to neonatal mice from day 5 to day 15 (**Figure 2**). The persistence of lactobacilli in suckling mice and administration route were determined in previous experiments (**Supplementary Figure S1**). Mice were challenged with *G. duodenalis* WB6 trophozoites at day 10 by intragastric gavage (1 × 10^5^ trophozoites) and sacrificed at day 16 (**Figure 2**). In preliminary experiments, we showed that this parasite strain was able to persist and colonize in OF1 suckling mice model, the peak of trophozoite load being reached at day 16 (i.e., 6 days post-inoculation) (**Supplementary Figure S2**). On the other hand, we demonstrated that lactobacilli strains can persist in the gut up to 3–4 days after intragastric gavage.

**FIGURE 3:**
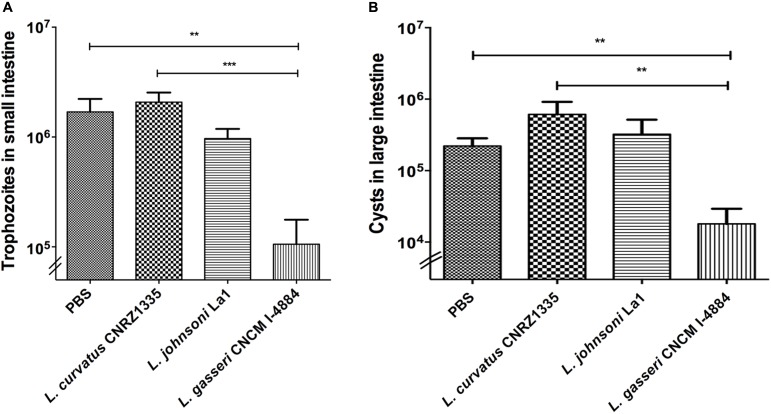
*In vivo* activities of *Lactobacillus gasseri* CNCM I-4884 and La1 lactobacilli strains against *G. duodenalis.* **(A)** *G. duodenalis* trophozoites enumeration after administration to animals. Suckling mice received either PBS (*n* = 8), La1 (*n* = 12), *L. gasseri* CNCM I-4884 (*n* = 10) or *L. curvatus* CNRZ1335 (*n* = 9) by intragastric gavage (5 × 10^8^ CFU/mice) daily from day 5, before inoculation with *G. duodenalis* WB6 trophozoites (10^5^ trophozoites per animal) at day 10. Gavages were performed until day 15. Small intestines were resuspended in PBS and trophozoites were counted using a hemocytometer. Values are mean ± SEM; *p* < 0.05. **(B)** *G. duodenalis* cysts counting in colon and caecum. Suckling mice received either PBS/glycerol 15%, La1, *L. gasseri* CNCM I-4884 or *L. curvatus* CNRZ1335 by intragastric gavage (5 × 10^8^ CFU/mice) daily from day 5 before inoculation with *G. duodenalis* WB6 trophozoites (10^5^ trophozoites per animal) at day 10. Gavages were performed until day 15 (*n* = 8–12/group). Colons and caeca were resuspended in 5 ml of ice-chilled 2.5% potassium dichromate and cysts were counted using a hemocytometer. Values are mean ± SEM. ^∗∗^*p* ≤ 0.01; ^∗∗∗^*p* ≤ 0.001.

Mice were divided into four groups with a minimum of eight animals per group. The parasite burden in mice treated with PBS/glycerol was 20-fold higher than the initial dose showing that trophozoites were able to colonize, multiply, and persist in the small intestine. As shown in **Figure 3A**, mice treated with *L. curvatus* CNRZ1335 were not protected against *G. duodenalis* challenge as they present a similar profile in trophozoite colonization and proliferation. In contrast, mice treated with *L. gasseri* CNCM I-4884 exhibited an 18-fold reduction in the trophozoites load in the small intestine (∼93%) (*p* < 0.001) compared to the PBS/glycerol group (**Figure 3A**). Mice fed with the probiotic strain La1 exhibited a twofold reduction in the trophozoites load compared to controls (∼43%). Interestingly, *L. gasseri* CNCM I-4884 was more efficient than La1 in preventing *G. duodenalis* proliferation *in vivo*.

Cysts enumerations were performed in both colon and caecum. We observed a significant reduction (-81%) (*p* < 0.01) in cyst formation in groups treated with *L. gasseri* CNCM I-4884 compared to both PBS/glycerol and *L. curvatus*-treated groups. No significant reduction was observed in the group treated with either La1 or *L. curvatus* CNRZ1335 compared to control groups treated with PBS/glycerol (**Figure 3B**).

## Discussion

Several studies have demonstrated that probiotics might play key roles against enteropathogens including intestinal prototozan parasites (see Travers et al., 2011, for a review). In the last 10 years, several lactobacilli strains have been studied for their ability to prevent the establishment of *G. duodenalis in vivo* and to reduce the severity of giardial infections (Humen et al., 2005; Shukla et al., 2008; Goyal et al., 2011; Goyal and Shukla, 2013). In some instances, *in vitro* assays were designed to decipher the molecular mechanisms involved in these protective effects. For the probiotic strain La1 of *L. johnsonii*, previously known to antagonize *Giardia* growth *in vitro* and *in vivo,* Perez and collaborators established the presence of an active principle in the supernatant of this probiotic strain (Perez et al., 2001; Humen et al., 2005). More recently, we undertook the molecular characterization of this active principal from La1 and discovered at least one possible mechanism of action: the involvement of bacterial BSH-like activities, that would mediate anti-giardial effect by generating deconjugated bile salts (toxic for the parasite) from non-toxic conjugated bile salts (Travers et al., 2016). BSH are common in *Lactobacillus* and *Bifidobacterium*, playing an important role for colonization and persistence in the gut (Tanaka et al., 1999; Begley et al., 2006; Denou et al., 2008). Therefore, we looked for other probiotic strains (closely related or not to La1) exhibiting anti-giardial activities. We thus selected 29 lactobacilli strains from diverse origins and screened their supernatants for their putative anti-giardial activities *in vitro* while, in parallel, we analyzed their BSH activities and properties. Among the supernatants issued from the 29 analyzed strains, 19 were found to display anti-parasitic activities on fresh trophozoites *Giardia* cultures. Interestingly, these 19 strains also displayed the highest BSH scores, indicating a correlation between these two properties. These results thereby suggest that a screening method based on BSH activities may predict potential anti-giardial activity in lactobacilli. Interestingly, *L. gasseri* CNCM I-4884 and La1 strains (which killed 100% of trophozoites *in vitro*) displayed both TDCA and GDCA specific BSH activities. We therefore propose that this dual or combined BSH activity would contribute to reinforce the release of deconjugated bile salt toxic for *Giardia*, from a larger panel of substrates. However, no clear relationships appeared between the observed anti-giardial activity and the environmental origins of the lactobacilli isolates (**Table 1**).

*Lactobacillus gasseri* CNCM I-4884 and La1 were subsequently administrated to OF1 suckling mice to assess their antagonistic effects *in vivo*. *L. curvatus* CNRZ1335, which displayed neither anti-giardial activity nor BSH activities *in vitro*, was used as a negative control. High trophozoites burden in controls showed efficient colonization and proliferation of the *G. duodenalis* WB6 in the OF1 mice (Shukla et al., 2008). Mice treated with *L. gasseri* CNCM I-4884 exhibited a dramatic reduction of viable trophozoites in their small intestines, 6 days post-challenge. La1 strain supplementation also lead to a reduction of trophozoites in mice intestines, but to a much lesser extent than *L. gasseri* CNCM I-4884. Previous studies have reported anti-giardial properties of lactobacilli strains. For instance, *L. casei* MTCC1423 and LGG strains reduced the duration and the severity of *G. duodenalis* infection (Portland strain) in C57BL/6 mice in 7–14 days (Shukla et al., 2008; Goyal et al., 2011) and La1 antagonized *Giardia* (WB6) in Mongolian gerbils in 7–21 days (Humen et al., 2005). However, no correlation between the anti-giardial effects and a BSH-like activity was established in these studies. Strikingly, in the current study, administration of *L. gasseri* CNCM I-4884 led to a stronger reduction of trophozoites load in a short period of time. Cyst formation is another important indication of the control of infectious parasite development (Lujan et al., 1997). A treatment capable of preventing trophozoite differentiation into cysts is indeed essential to minimize their spreading in the environment via animals’ feces. We observed that mice fed with *L. gasseri* CNCM I-4884 exhibited a dramatic reduction of cyst excretion 6 days post-challenge compared to untreated infected mice. This is in concordance with previous results showing that other lactobacilli such as *L. casei* MTCC 1423 and LGG were effective in eliminating cysts after 7 days of treatment. In contrast, no cyst reduction was observed in mice treated with La1 although its ability to eliminate cysts has been previously reported in mongolian gerbils (Humen et al., 2005). The differences in terms of *in vivo* efficiency between *L. gasseri* CNCM I-4884 and La1, both BSH-positive strains, can be explained by either a better hydrolysis of conjugated bile salts *in vivo*, a higher ability for competing for biological niches in the small intestine, and a better persistence in the gut.

To date, the underlying mechanisms involved in the antagonistic effect of lactobacilli against *Giardia* is an emerging field. Besides the role of BSH-like activities that we have previously reported for La1 (Travers et al., 2016), and now for other *Lactobacillus* strains, other mechanisms have been identified. Among them, the role of anti-microbial peptides in the anti-giardial activity of lactobacilli has been explored. Indeed, when administered orally, P106, a derived bacteriocin isolated from *L. acidophilus*, was shown to reduce the trophozoite burden in mice at high concentrations (Amer et al., 2014). However, the ability to produce anti-microbial peptides is strain dependent and highly variable and this cannot be extrapolated to all anti-giardial strains. Also, probiotic lactobacilli are known to enhance the mucosal immune system, which participate in clearing enteropathogens from the gut (Howarth and Wang, 2013). The immunomodulatory properties of lactobacilli have also been proposed to explain their anti-giardial properties (Goyal and Shukla, 2013; Allain et al., 2017; Barash et al., 2017; Bartelt et al., 2017; Fink and Singer, 2017). In our study, the local and systemic response to *Giardia* infection has not been assessed due to the fact that suckling mice have an immature immune system (Basha et al., 2014).

While the anti-giardial properties of lactobacilli appear to be multifactorial, there is a clear contribution of BSH-activities. However, further experiments are necessary to investigate the potential of BSH in treating giardiasis, including the direct effect of BSHs *in vitro* and *in vivo*. Knockout mutant strains for all BSH genes (several BSH genes are commonly found in lactobacilli) are still needed to evaluate the relative contribution of each one of these enzymes. To summarize, this study shows BSH activity as a key screening parameter to identify anti-*Giardia* lactobacilli strains. In addition, *L. gasseri* CNCM I-4884, which displayed both high anti-giardial and BSH activities *in vitro*, antagonizes *Giardia* survival in OF1 mice. This study represents a significant step toward the development of new prophylactic strategies, with both human and veterinary applications.

## Author Contributions

IF, BP, PG, TA, and LB-H conceived and designed the study. TA, BP, SC, and MT performed all the experiments. M-AT, IV, and PL discussed the experiments and results. TA, IF, and LB-H wrote the manuscript.

## Conflict of Interest Statement

The authors declare that the research was conducted in the absence of any commercial or financial relationships that could be construed as a potential conflict of interest.

## Supplementary Material

FIGURE S1 Persistence of *L. johnsonii* La1 strain (Ery^r^) in OF1 suckling mice. Each mouse received a single administration of 5 × 10^8^ CFU of *L. johnsonii* La1 Ery^r^ either by oral gavage (days 1, 2, and 3; *n* = 4) or intragastric gavage (days 1, 2, 3, and 4; *n* = 4). *L. johnsonii* La1 was transformed with a plasmid harboring an erythromycin (Ery)-resistance gene as described previously (Allain et al., 2016). Values are in mean ± SEM.

FIGURE S2 Kinetics of infection of *G. duodenalis* strain WB6 in OF1 suckling mice. **(A)** *G. duodenalis* trophozoites enumeration in small intestine after single gavage (day 0). Trophozoite burden was measured at days 3, 6, 8, 10, 12, 15, and 18 (*n* = 10). Small intestines were resuspended in PBS and trophozoites were counted using a hemocytometer **(B)** *G. duodenalis* cysts enumeration in large intestine after single gavage (day 0). Cysts were measured at days 3, 6, 8, 10, 12, 15, and 18 (*n* = 10). Large intestines were resuspended in 2.5% Potassium dichromate and cysts were counted using a hemocytometer. Values are in mean ± SEM.
