# Supplementary figures and images for: Bile Salt Hydrolase Activities: A Novel Target to Screen Anti-Giardia Lactobacilli?
Source: Front Microbiol. 2018 Feb 8;9:89. doi: 10.3389/fmicb.2018.00089 (PMC5809405; doi:10.3389/fmicb.2018.00089)

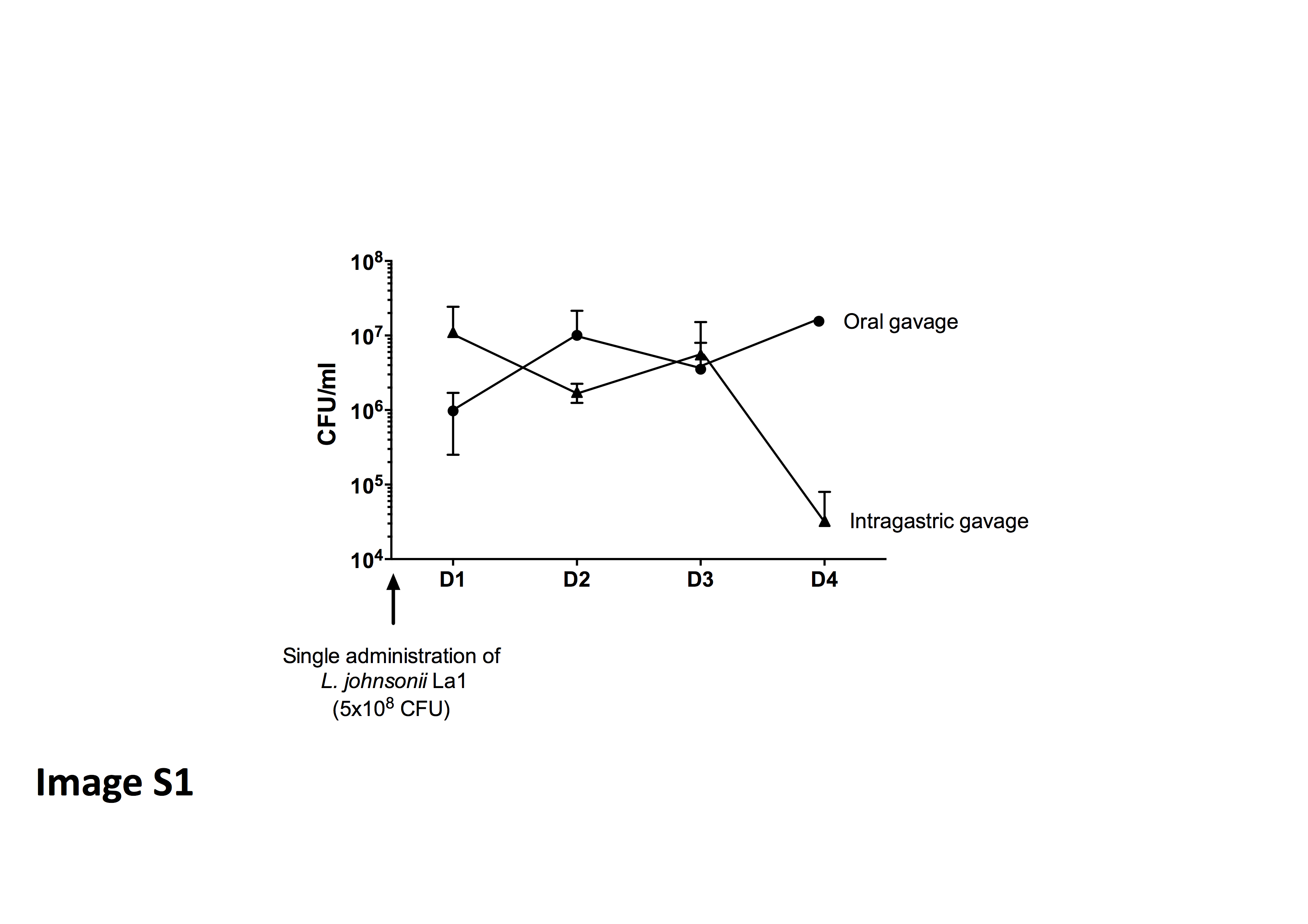

Supplement: FIGURE S1 — Persistence of L. johnsonii La1 strain (Eryr) in OF1 suckling mice. Each mouse received a single administration of 5 × 10^8^ CFU of L. johnsonii La1 Eryr either by oral gavage (days 1, 2, and 3; n = 4) or intragastric gavage (days 1, 2, 3, and 4; n = 4). L. johnsonii La1 was transformed with a plasmid harboring an erythromycin (Ery)-resistance gene as described previously (Allain et al., 2016). Values are in mean ± SEM. [file Image_1.TIFF]

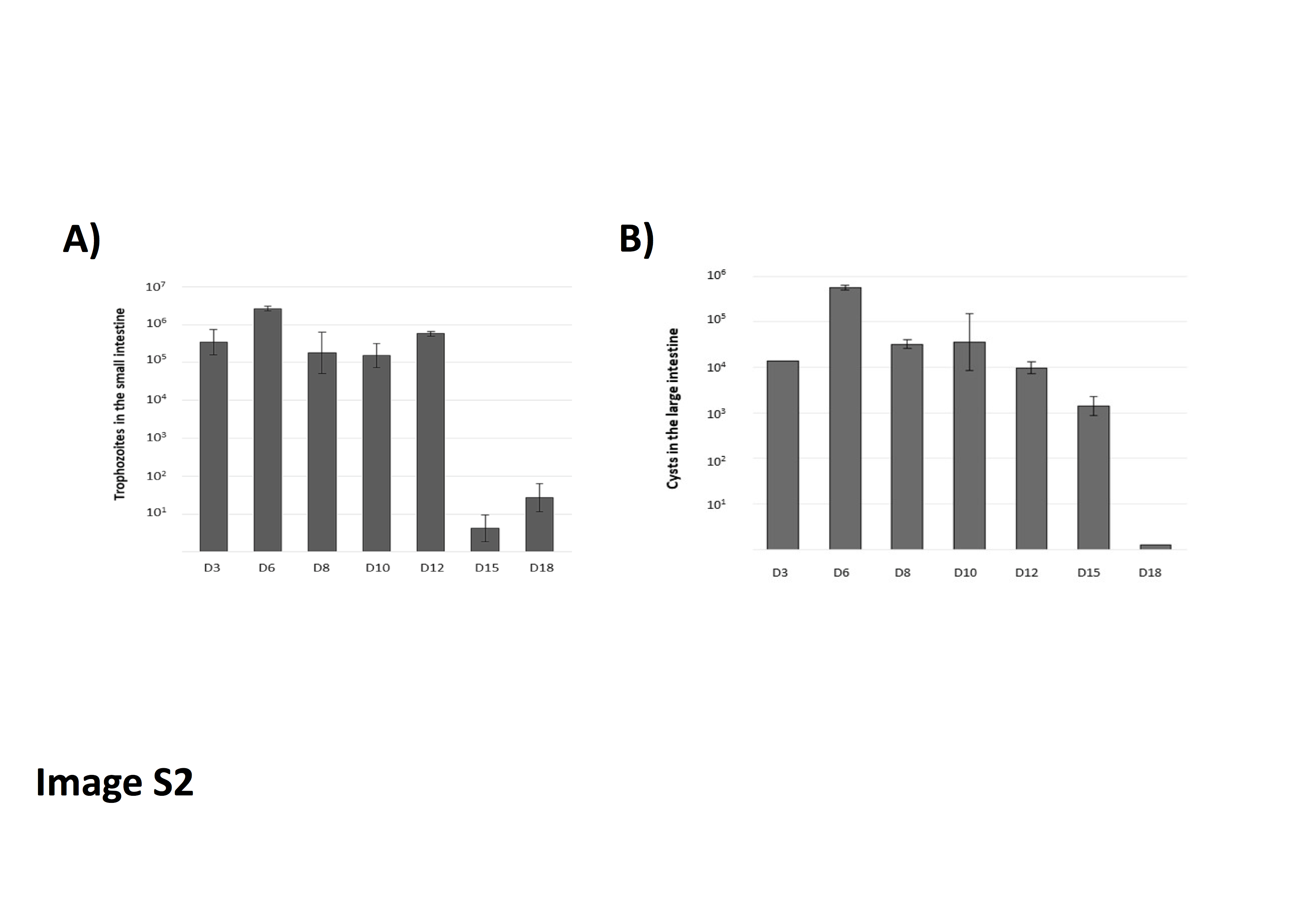

Supplement: FIGURE S2 — Kinetics of infection of G. duodenalis strain WB6 in OF1 suckling mice. (A) G. duodenalis trophozoites enumeration in small intestine after single gavage (day 0). Trophozoite burden was measured at days 3, 6, 8, 10, 12, 15, and 18 (n = 10). Small intestines were resuspended in PBS and trophozoites were counted using a hemocytometer (B) G. duodenalis cysts enumeration in large intestine after single gavage (day 0). Cysts were measured at days 3, 6, 8, 10, 12, 15, and 18 (n = 10). Large intestines were resuspended in 2.5% Potassium dichromate and cysts were counted using a hemocytometer. Values are in mean ± SEM. [file Image_2.TIFF]
